# Rare widespread dissemination of cervical high-grade squamous intraepithelial lesion with microinvasive squamous cell carcinoma: a case report

**DOI:** 10.3389/fonc.2025.1654368

**Published:** 2025-08-13

**Authors:** Shijie Liu, Dan Liu

**Affiliations:** ^1^ Department of Pathology, Dalian Women and Children's Medical Group, Dalian, Liaoning, China; ^2^ Department of Pathology, The First Affiliated Hospital of Dalian Medical University, Dalian, Liaoning, China

**Keywords:** high-grade squamous intraepithelial lesion, squamous cell carcinoma, superficial spreading, endometrium, fallopian tube, ovary

## Abstract

**Background:**

Cervical high-grade squamous intraepithelial lesion (HSIL), a precancerous condition, can progress to cervical squamous cell carcinoma (CSCC), the most prevalent histological subtype of cervical cancer. Although CSCC most commonly metastasizes via lymphatic or hematogenous routes, contiguous superficial spread to the endometrium, fallopian tubes, and ovaries is rare.

**Case presentation:**

A 61-year-old postmenopausal woman was referred to our hospital for further evaluation after a positive HPV-16 test and normal ThinPrep Cytologic Test (TCT) results during a routine health examination at an external institution two weeks earlier. Histopathological examination of colposcopy-guided biopsies confirmed chronic cervicitis with HSIL. Notably, the serum squamous cell carcinoma antigen (SCC-Ag) level was markedly elevated (32.20 ng/mL). Transvaginal color Doppler ultrasonography revealed a cystic mass in the right pelvic region. Intraoperative laparoscopic findings included a tortuous, thickened right fallopian tube with fimbrial occlusion. Gross pathological examination revealed an irregular grayish-white endometrial lesion measuring 2.5*2.0 cm. The right fallopian tube exhibited focal dilation, measuring 1.6 cm in diameter. No gross abnormalities were detected in the right ovary. Final histopathology confirmed extensive cervical HSIL (CIN III) with multifocal stromal invasion (maximum depth: 4 mm), which involved the endometrium, right fallopian tube mucosa, and an ovarian inclusion cyst on the ipsilateral side.

**Conclusion:**

Cervical HSIL/SCC may exhibit superficial upward extension to the endometrium and, in rare cases, can involve the ovaries. Although rare, this clinical entity warrants increased clinical vigilance. Currently, no standardized management guidelines exist for this distinct metastatic pattern, and emerging evidence suggests a multifactorial pathogenesis. These findings underscore the need for enhanced early detection and preventive strategies.

## Introduction

Cervical cancer remains a major global health burden, ranking as the fourth most prevalent malignancy in women worldwide (1). Among all histological subtypes, SCC is the most prevalent, typically arising from its precursor lesion, HSIL. The disease typically extends inferiorly into the vagina or laterally infiltrates the parametrial tissue, with potential metastasis via lymphatic spread to regional and occasionally distant lymph nodes or hematogenous dissemination (2). An exceedingly rare pattern involves superficial endometrial spread, with even rarer proximal extension to the fallopian tubes and ovaries. Due to the exceptional rarity of this condition, optimal staging criteria, treatment strategies, and prognostic outcomes remain poorly defined. Herein, we present the first reported case of cervical HSIL/SCC with superficial spread to the endometrium, fallopian tubes, and ovaries, and discuss potential mechanisms underlying this unusual dissemination pattern.

## Case presentation

A 61-year-old postmenopausal woman had tested positive for HPV-16 during a routine examination at an external hospital two weeks prior; however, the ThinPrep Cytologic Test (TCT) revealed no significant abnormalities. She was subsequently referred to our hospital for further evaluation, where colposcopy-guided biopsy confirmed chronic cervicitis with HSIL. The patient had been postmenopausal for 10 years, with an unremarkable reproductive history and no history of hypertension, diabetes, tobacco use, alcohol consumption, or family history of malignancy. She had a history of Behçet’s disease, which had been diagnosed two years earlier. Serological testing revealed IgG-type antinuclear antibody (ANA) positivity, and she received oral methylprednisolone with gradual dose adjustments. The patient had undergone left-eye cataract surgery in 2024. Pelvic examination revealed vulvar, vaginal, and cervical atrophy. The uterus was anteverted and nontender, with right adnexal thickening but no significant bilateral tenderness. Transvaginal ultrasound showed an indistinct right ovary and an irregular hypoechoic mass (41*29 mm) in the right pelvis, with incomplete septations and punctate blood flow signals within the septa. The inner wall and septa were irregular, with tiny hyperechoic protrusions. No significant abnormalities were found on the remaining imaging. She underwent laparoscopic total hysterectomy with bilateral salpingo-oophorectomy, without lymph node dissection. Intraoperative findings revealed a normal-sized, smooth-surfaced uterus; atrophic endometrium and bilateral ovaries; no cervical induration or friability; and a tortuous, thickened right fallopian tube with fimbrial occlusion. The preoperative serum tumor markers, including SCC-Ag (10.60 ng/mL), CA125 (73 U/mL), CA19-9 (124 U/mL), and HE4 (143 pmol/L), were markedly elevated. The patient recovered well and was discharged on the fourth postoperative day.

We obtained an intact uterine specimen measuring 5.0*3.5*2.5 cm. The serosal surface appeared unremarkable, with no gross abnormalities. The endometrium was 0.2 cm thick, and the myometrium measured up to 1.3 cm in thickness. The ectocervix measured 2.0*1.5 cm, with smooth cervical and endocervical mucosa. The right fallopian tube showed focal dilatation (maximum diameter: 1.6 cm), while the left adnexa and right ovary appeared grossly normal. Representative sections from all relevant areas were submitted for histopathological examination.

**Figure 1:**
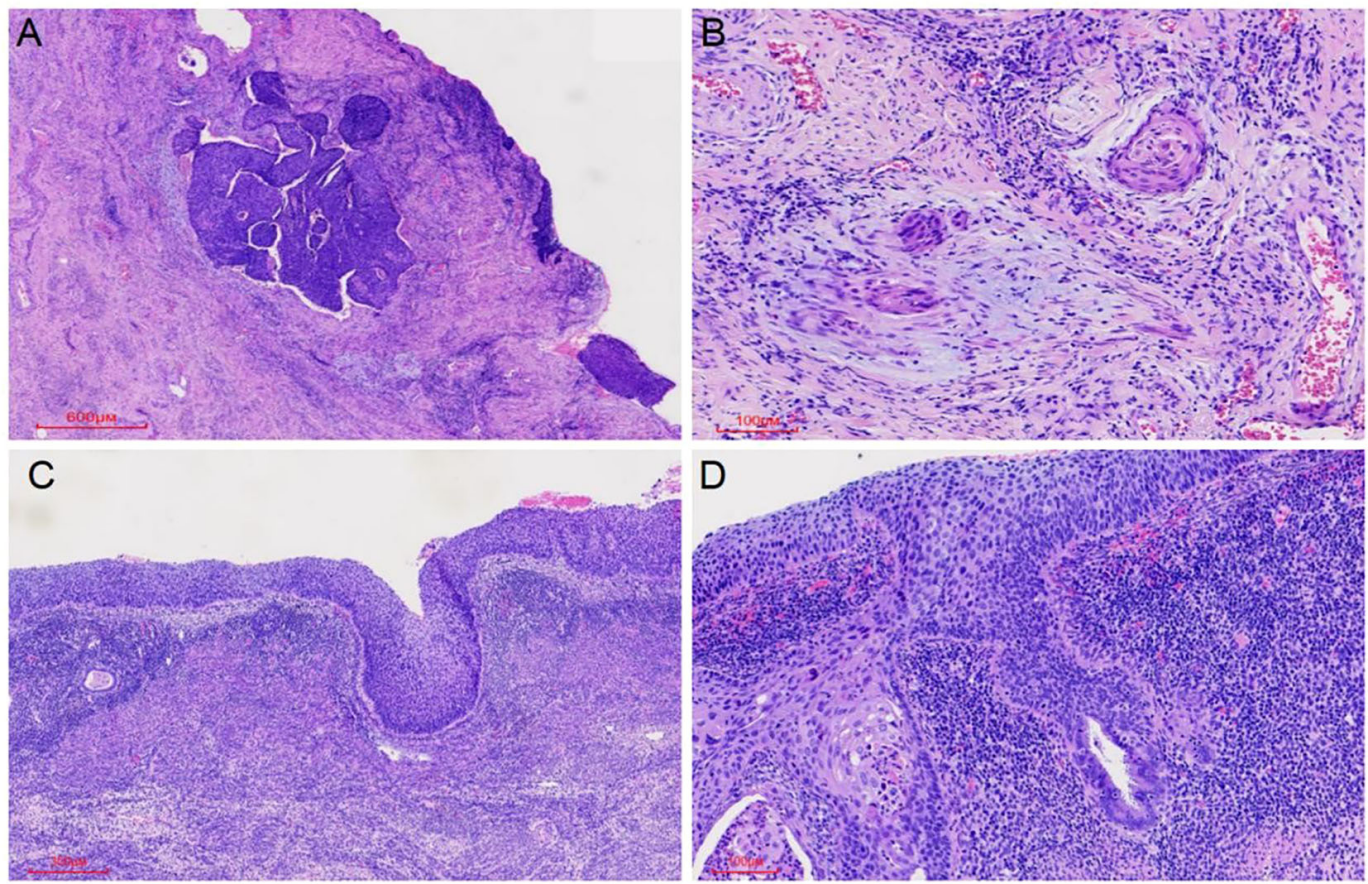
HSIL (CIN III) involving the cervical glands was observed, along with microinvasive foci (maximum depth 4 mm) **(A, B)** and extension to the endometrial glands and surface epithelium **(C, D)**.

**Figure 2:**
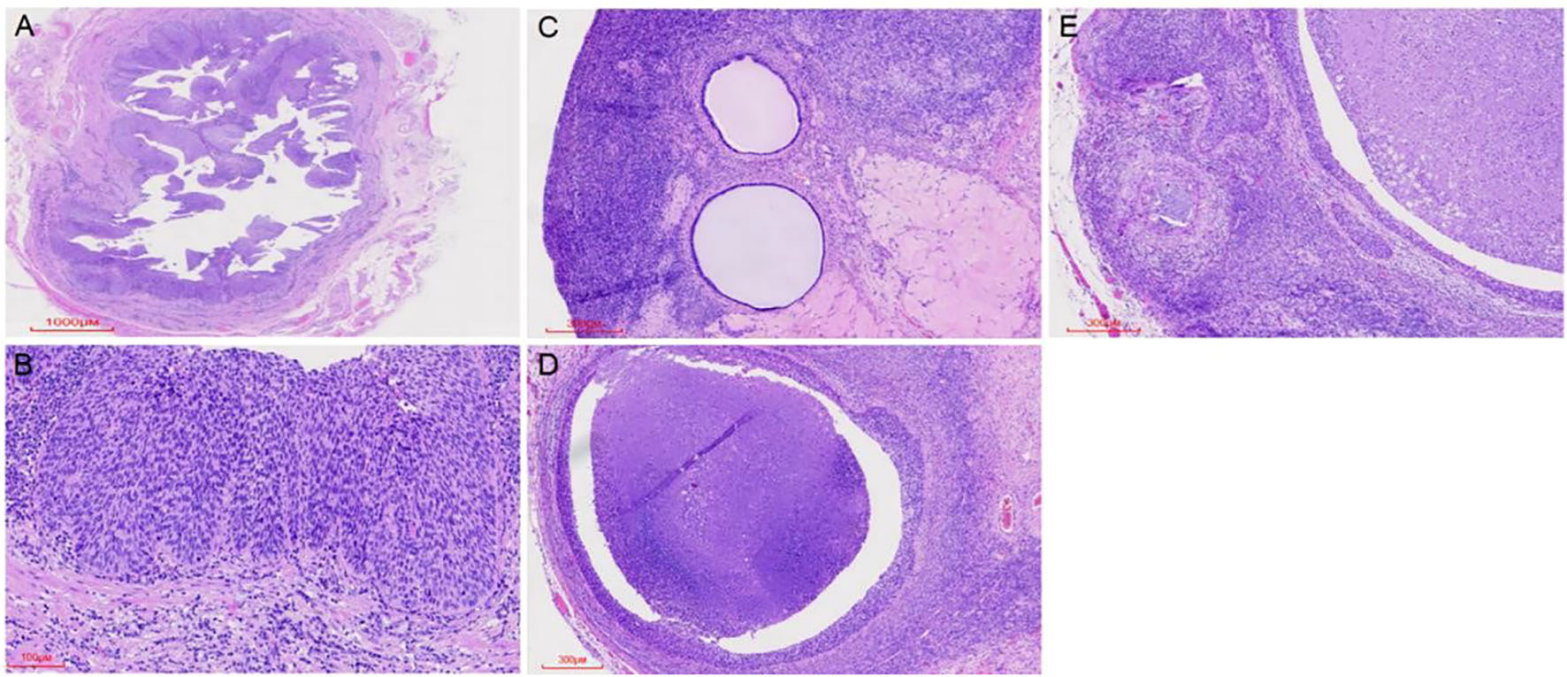
HSIL (CIN III) was observed replacing the normal tubal epithelium **(A, B)**. HSIL (CIN III) involved inclusion cysts, with associated microinvasive foci. Cortical inclusion cysts **(C)** were identified, along with round to irregular nests of varying sizes containing necrotic debris in the ovary **(D)**. Microinvasive foci were also detected in the ovary **(E)**.

Microscopic examination of the cervix showed extensive HSIL with glandular involvement and multifocal stromal invasion (**Figures 1A, B**), measuring up to 4 mm in depth. The endometrium appeared atrophic. The endometrial tissue was extensively replaced by HSIL, which involved both the glands and surface epithelium (**Figures 1C, D**). Unexpected HSIL involvement was identified in the right fallopian tube mucosa during routine adnexal examination (**Figures 2A, B**). Notably, HSIL with stromal microinvasion was identified in the right ovary, manifested as round-to-irregular nests accompanied by cysts containing necrotic debris. Adjacent inclusion cysts were observed, suggesting potential HSIL involvement (**Figures 2C-E**). No evidence of lymphovascular or perineural invasion was found.

**Figure 3:**
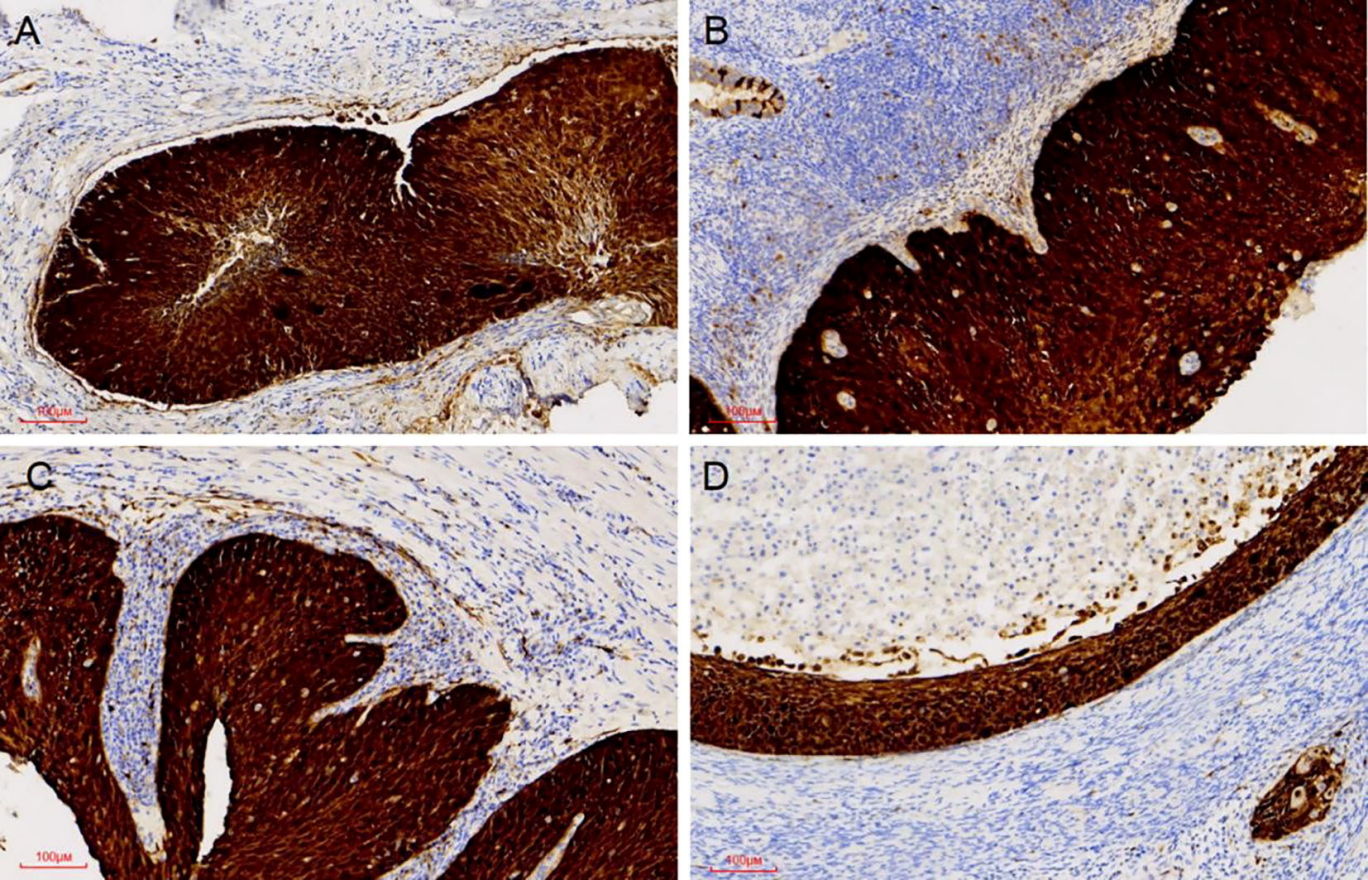
P16 expression was positive in all examined sites. Diffuse P16 immunostaining was observed in the tumour cell nuclei of the cervix, the endometrium, the right fallopian, and the right ovary **(A-D)**.

**Figure 4:**
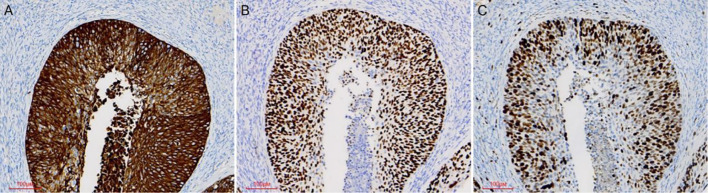
Immunohistochemical staining was performed on ovarian tumor samples. Ovarian tumor cells exhibited positivity for the CK5/6 marker **(A)**. Tumor cells showed immunoreactivity for the P40 marker **(B)**. The Ki67 proliferative index was approximately 80% **(C)**.

Pathological examination demonstrated tumor components exhibiting shared cytological features in the right ovary, ipsilateral fallopian tube, and endometrium, which were consistent with superficial CSCC. Although no similar cases were reported in the literature, the morphological similarities among the tumors suggested a probable origin from disseminated primary CSCC. For diagnostic confirmation, systematic immunohistochemical staining was conducted on tissue samples obtained from each site. Immunohistochemical analysis revealed diffuse p16 positivity in tumor cells isolated from the endometrium, fallopian tube, and ovary (**Figure 3**). Additionally, ovarian tumor cells showed positivity for CK5/6 and p40, accompanied by a Ki-67 proliferation index of approximately 80% (**Figure 4**). During the 4-month postoperative follow-up, the patient maintained clinical stability without complications or evidence of tumor recurrence. Prospective long-term follow-up is being conducted to monitor clinical outcomes.

## Discussion and conclusions

Superficially spreading SCC is defined as a subtype of CSCC characterized by superficial growth along the endometrial surface, accompanied by replacement of the endometrial epithelium (2). This variant is extremely rare. In the reported cases, the endometrium was the predominant site of involvement, typically presenting as isolated carcinoma *in situ* without invasive growth. However, very few reports describe involvement of unilateral or bilateral fallopian tubes and/or ovaries (3).

The 2018 FIGO staging system for cervical cancer classified microinvasion (depth ≤3 mm) as stage IA1, which was typically associated with an excellent prognosis (4). However, this case exhibited rare biological behavior, including synchronous involvement of the endometrial cavity, right fallopian tube, and ipsilateral ovarian surface—a presentation not described by the current FIGO staging system, which complicated clinical management (5). These findings suggested that tumor heterogeneity and field effects might necessitate additional stratification criteria. Current guidelines for FIGO IA1 recommended conservative excision provided that margins were negative (4). However, in cases such as the present one, the risk of residual disease might have been underestimated. We recommend preoperative evaluation of the endometrium and adnexa using advanced diagnostic modalities to identify pathological features, particularly multifocal lesions or microinvasion, thereby optimizing clinical decision-making for radical hysterectomy to improve survival outcomes.

Several studies have examined this rare superficial spreading pattern. Potential risk factors comprise HPV infection, long-term estrogen use, vitamin A deficiency, advanced age, pyometra, and radiotherapy (6). Among these, high-risk HPV infection is responsible for 90%-95% of SCC cases, indicating that persistent infection plays a pivotal role in carcinogenesis (7). Furthermore, persistent HPV infection increases the risk of cervical lesion progression and post-treatment recurrence, warranting risk-stratified monitoring and targeted intervention (8).

Ovarian metastasis is observed in only 0.4-1.3% of CSCC patients. The potential routes of metastasis comprise direct invasion, lymphatic spread, hematogenous dissemination, and trans-tubal migration (9). Tong et al. reported that, five years post-hysterectomy, patients with microinvasive stage IA1 CSCC without lymphovascular space invasion (LVSI) showed recurrence limited to the ovaries, with no endometrial or tubal involvement (10). Zhang et al. described a rare case of stage IA1 CSCC with extensive metastasis affecting the endometrium, bilateral fallopian tubes, and right ovary, accompanied by LVSI (9). Taken together, these reports indicate potential metastatic mechanisms, such as lymphovascular invasion and trans-tubal migration. Notably, Wegscheider et al. reported a case of CSCC with HSIL in the fallopian tube fimbria but no other neoplastic involvement, illustrating a skip metastasis pattern (11). In contrast, our case exhibits upward progression from HSIL/CIN III to the uterine corpus, followed by tubal and ovarian involvement. Histopathological examination showed no metastatic carcinoma in the uterine corpus or fallopian tube, but only focal stromal microinvasion in the ovarian stroma. This growth pattern suggests that contiguous tumors propagate along the anatomical pathway.

In rare cases, cervical HSIL may exhibit superficial upward extension along the endometrial surface, with continuous spread to the fallopian tubes and ovaries, forming a distinctive ‘creeping’ lesion pattern. Throughout this progression, the lesions maintain intact basement membrane integrity without stromal invasion (12). At the genomic level, Kushima et al. identified five similar cases exhibiting shared allelic losses at 6p, 6q, 11p, and 11q in both cervical and upper genital tract lesions. These findings support a monoclonal origin, suggesting that the SCC originated from a single progenitor cell and subsequently underwent clonal expansion during cephalad migration (13). Ishida et al. demonstrated that CD138-mediated regulation of cell-matrix interactions orchestrates a unique collective migration pattern along mucosal surfaces, which differs fundamentally from conventional infiltrative dissemination at both cellular and molecular levels (2). This pattern suggests that tumor cells may migrate continuously via a crawling-like mechanism along tissue surfaces. Notably, Zhang et al. first reported a case of superficially invasive CSCC metastasizing to the wall of an ovarian endometrioma, with no evidence of malignancy in the ovary, fallopian tube, or other specimens, suggesting that the cancer cells may have spread along the endometriotic lesions via superficial crawling (14).

Kurman et al. first proposed the “tubal-ovarian hypothesis” suggesting that some ovarian serous carcinomas might originate not from the ovarian epithelium itself but from the fimbrial end or mucosal epithelium of the fallopian tube, subsequently implanting on the ovarian surface, where both lesions share identical mutational profiles, including TP53 mutations (15). Although the tubal-ovarian hypothesis primarily implicates the fallopian tube as the origin, ovarian cortical inclusion cysts (CICs) could serve as potential intermediaries through the following mechanisms. First, the ovarian surface epithelium lining CICs can undergo metaplastic transformation into fallopian tube–like ciliated epithelium, thereby generating a local microenvironment of Müllerian metaplasia within the ovary. These metaplastic epithelial cells, upon acquiring specific genetic mutations (e.g., TP53 or BRAF/KRAS alterations), can progress to low-grade serous carcinoma (LGSC) (15). Alternatively, the “homing” effect involves the shedding of precancerous cells from the fimbrial epithelium, which can migrate with tubal fluid to the ovarian surface and become incorporated into CICs, potentially initiating secondary tumor formation (16). Genomic tracing studies have further corroborated this observation (17). Furthermore, ovarian stromal-derived factors (e.g., CXCR4, IL-8, and TNF-α) have been demonstrated to facilitate this homing process (18). The superficial spread of SCC to the endometrium and upper genital tract represents a rare but well-documented phenomenon. Collectively, these findings suggest that synergistic interactions among multiple factors drive the pathogenesis of this rare disease.

This study has several limitations. First, as a case report, our observations cannot be generalized to a broader population. Given the rarity and complexity of this phenomenon, future larger cohort studies are needed to elucidate the pathological mechanisms, prognostic factors, and optimal management strategies. Second, although immunohistochemical analysis supports the diagnosis, the proposed mechanisms of tumor spread remain speculative because of the lack of molecular profiling, comprehensive HPV genotyping of all involved tissues, and lymph node dissection, highlighting the need for further investigations to provide more precise guidance for clinical decision-making. Finally, owing to the relatively short follow-up period in this case, the relationship between superficially spreading SCC and long-term prognosis remains uncertain.

The presence of endometrial and adnexal involvement in superficially spreading SCC may indicate a poor prognosis. Therefore, comprehensive preoperative evaluation, ideally conducted through multidisciplinary assessment, is crucial for assessing endometrial and adnexal status prior to hysterectomy. Current literature suggests that most cases present as widely invasive cervical squamous cell carcinomas with frequent proximal uterine involvement. The findings of this study demonstrate that metastatic lesions may exhibit unpredictable biological behavior. Notably, superficial spread of cervical cancer to the proximal uterus and adnexa represents an exceptionally rare clinical phenomenon. This suggests that in selected cases, particularly those with extensive or recurrent HSIL, multifocal microinvasive SCC, or resistance to conservative treatment, radical hysterectomy may be considered a definitive therapeutic option. Additionally, greater focus on early detection, prevention, and evidence-based management guidelines is warranted.

## Data Availability

The original contributions presented in the study are included in the article/supplementary material. Further inquiries can be directed to the corresponding author.
